# A new method to measure cell metabolism of rare cells *in vivo* reveals a high oxidative phosphorylation dependence of lung T cells

**DOI:** 10.1111/imcb.70018

**Published:** 2025-04-23

**Authors:** Aristeidis Roubanis, Morgane Hilaire, Morgane Le Teuff, Odile Devergne, Tim Sparwasser, Luciana Berod, Benoît L Salomon

**Affiliations:** ^1^ Centre d’Immunologie et des Maladies Infectieuses (CIMI‐Paris), INSERM, CNRS, Sorbonne Université Paris France; ^2^ Toulouse Institute for Infectious and Inflammatory Diseases (Infinity) INSERM – CNRS – University Toulouse III Toulouse France; ^3^ Institute of Medical Microbiology and Hygiene and Research Center for Immunotherapy (FZI) University Medical Center, Johannes Gutenberg‐University Mainz Mainz Germany

**Keywords:** Cell metabolism, Foxp3, *in vivo* SCENITH, lung T cells, Treg

## Abstract

Regulation of cellular metabolism is a central element governing the fate and function of T cells. However, the *in vivo* metabolic characteristics of rare cells, such as nonlymphoid tissue T cells, are poorly understood because of experimental limitations. Most techniques measuring cell metabolism require large cell numbers. The recent SCENITH method allows for studying the metabolism of rare cells by flow cytometry. However, this technique requires cells to be isolated and cultured *ex vivo*, which may alter their metabolism. Here, we propose a new experimental approach, called *in vivo* SCENITH, to investigate the cellular metabolism of T cells *in vivo* at a steady state in the spleen and lungs. For this purpose, we administered the metabolic modulators directly in mice, instead of applying these reagents *ex vivo*, as in the classical SCENITH method. Whereas *ex vivo* manipulation impacted the viability and phenotype of T cells, this toxic effect was not observed in the *in vivo* SCENITH. We observed that conventional and regulatory T cells shared similar metabolic profiles. Importantly, whereas spleen T cells used both oxidative phosphorylation and glycolysis, the metabolism of T cells in the lungs was mainly based on oxidative phosphorylation. Finally, metabolic inhibitors that interfere with protein translation and energy availability downregulated Foxp3 expression in regulatory T cells. These results describe an expansion of SCENITH that allows to measure the metabolic profile of rare cells *in vivo*, revealing a high dependence on oxidative phosphorylation of lung T cells.

## Graphical abstract

In this article, we adapted the SCENITH assay to measure the metabolism of rare cells *in vivo.* We observed that, under steady‐state conditions, lung T cells were highly dependent on oxidative phosphorylation for energy production. Further, metabolic inhibitors that interfere with protein translation and energy availability downregulated Foxp3 expression in regulatory T cells, which was better revealed using the classical *ex vivo* SCENITH assay.

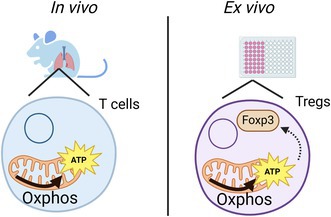

## INTRODUCTION

The study of cellular metabolism of immune cells has been neglected for a long time. It is only in the last 15–20 years that this area of research, known as immunometabolism, has attracted the interest of a growing number of researchers. Seminal findings demonstrated that metabolic adaptation is an important component governing the function of T cells in immunity and diseases.^1^, ^2^ Modulating the metabolism of T cells may pave the way to discovering novel therapeutic strategies.

Different experimental approaches have been used to study immunometabolism.^3^ Important discoveries were made by investigating the outcome of altering metabolic pathways. This includes studies of patients with mutations in genes involved in metabolism, genetically modified mice or cells or studies using inhibitors of specific metabolic pathways.^4^ In addition, the metabolic activity of cells can be analyzed by multiple methods that are constantly evolving. Mass spectrometry can measure a large number of metabolites, identifying the activity of metabolic pathways in cell populations. Other omics data, including transcriptomics, proteomics or kinome data, provide more indirect but valuable complementary information.^5^, ^6^, ^7^ Extracellular measurements can be performed with specialized equipment, such as the SeaHorse analyzer that measures oxygen consumption or media acidification in response to metabolic inhibitors in culture to determine their oxidative phosphorylation (OxPhos) or glycolytic activity, respectively.

Recent advances in multiparametric flow cytometry introduced new perspectives in immunometabolism.^8^ The metabolic activity of cells can be assessed using antibodies detecting receptors or enzymes involved in metabolic pathways or fluorescent reagents that bind to metabolites. These analyses measure mitochondrial characteristics, metabolite uptake or expression levels of enzymes, but not directly the dependence of cells on metabolic pathways.^5^ A recent functional method, based on flow cytometry, called single‐cell energetic metabolism by profiling translation inhibition (SCENITH), was developed to address this limitation.^9^ This method is based on the concept that a large fraction of adenosine triphosphate produced by cells is used for protein synthesis. Thus, in most situations, cellular metabolic activity can be extrapolated from the level of translation. SCENITH is performed by measuring the level of puromycin (puro) incorporated in nascent proteins using a specific monoclonal antibody (mAb). Blocking metabolic pathways with specific inhibitors, such as glycolysis or OxPhos, induces a reduction of cellular adenosine triphosphate levels and therefore protein synthesis, which is quantified by flow cytometry using an anti‐puromycin mAb.^9^

The choice of the experimental approach and the method to analyze cell metabolism depend on the context and scientific question. Here, we wanted to analyze the metabolism of T cells, particularly Foxp3 regulatory T cells (Tregs), in lymphoid and nonlymphoid tissues. Two critical challenges were encountered. The first one was the rarity of these cells, especially for Tregs of nonlymphoid tissues. Consequently, metabolomics or proteomics by mass spectrometry methods, as well as SeaHorse‐type approaches cannot be used, as they require high cell numbers. SCENITH is well‐adapted for studying rare cells, as it relies on flow cytometry. The second challenge was the rapid metabolic adaptation of cells to microenvironmental changes. SCENITH has been used to study the metabolism of T cells after their extraction from tissues and short‐term culture during which puromycin and metabolic inhibitors were added.^10^, ^11^, ^12^, ^13^, ^14^ However, cellular metabolism may be modified during this procedure for several reasons. Preparing cells from tissues generates cellular stress, shifting their metabolic activity. Further, even if culture media can be optimized to closely mimic the plasma composition, *in vivo* microenvironments are not recapitulated. Key *in vivo* parameters such as complex cellular interactions, fluid dynamics and oxygen, metabolite or cytokine levels cannot be replicated in culture.^15^ Consequently, studying cell metabolism, either *ex vivo* or *in vitro*, may not reflect physiological contexts, highlighting the importance of technological advances to examine metabolism *in vivo*.

To address these two challenges, we evaluated the possibility of adapting the classical SCENITH assay toward an *in vivo* method, which was tested to assess the metabolic profile of conventional T cells (Tconvs) and Tregs in the spleen and lungs at the steady state. Mice were treated with metabolic inhibitors and puromycin instead of using these reagents *in vitro* as in classical SCENITH. The metabolism of Tregs and Tconvs was analyzed *in vivo*, identifying their reliance on glycolysis or OxPhos, and was compared with the one measured *ex vivo* using the classical SCENITH assay.

## RESULTS

### Splenic and lung T cells have distinct metabolic profiles measured *ex vivo*

**Figure 1:**
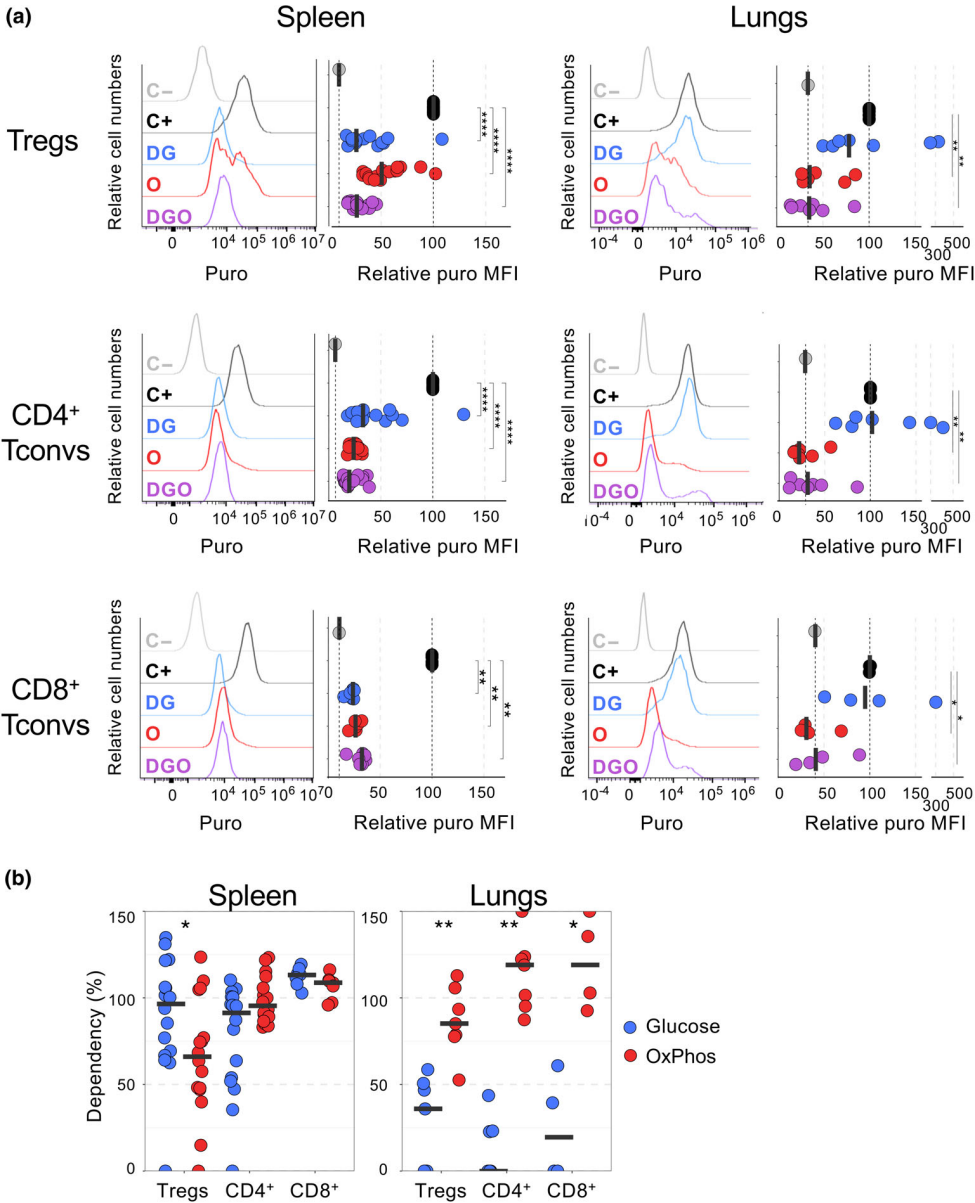
Splenic and lung T cells have distinct metabolic profiles *ex vivo*. The SCENITH assay was performed on spleen and lung cells cultured without puromycin (C–; puro), with puro alone (C+) or with puro and 2‐deoxyglucose (DG), puro and oligomycin (O) or puro and both DG and oligomycin (DGO). Puro incorporation in regulatory T cells (Tregs), CD4^+^ conventional T cells (Tconvs) and CD8^+^ Tconvs were analyzed by flow cytometry. **(a)** Representative puro staining (left panels) and puro mean fluorescent intensity (MFI) relative to the puro MFI of C+ control (right panels). **(b)** Glycolysis and oxidative phosphorylation (OxPhos) dependencies were calculated using puro MFI following the formula: 100 × ((((C+) − (DG))/(C+)) − (DGO)) for glycolysis dependency and 100 × ((((C+) − (O))/(C+)) − (DGO)) for OxPhos dependency. Each symbol represents individual mice and bars represent medians. Data are from at least three independent experiments. Number of mice per group: 16 for splenic CD4^+^ T cells, 7 or 8 mice for lung CD4^+^ T cells and splenic CD8^+^ Tconvs and 4 mice for lung CD8^+^ T cells. Statistical analysis was calculated using a Wilcoxon rank‐sum test. *P ≤ 0.05, **P ≤ 0.01, ***P ≤ 0.001 and ****P ≤ 0.0001.

To determine the metabolic profile of T cells from the spleen and lungs *ex vivo*, we used the protocol described in the classical SCENITH assay.^9^ Organs were dissociated and cell extracts were cultured for 50 min with 2‐deoxyglucose (DG or 2‐DG) or oligomycin (O) and then with puromycin. DG is an inhibitor of glycolysis resulting in the accumulation of 2‐deoxyglucose‐6‐phosphate, which in turn inhibits the function of hexokinase and glucose‐6‐phosphate isomerase. O is an inhibitor of OxPhos, which works by blocking the function of adenosine triphosphate synthase. The decrease of puromycin incorporation induced by these metabolic inhibitors allows us to calculate the dependence of cells on glucose and OxPhos. For splenic Tregs, puromycin's relative mean fluorescent intensity (puro MFI) was reduced by 50% when cells were treated with O and by 75–80% when treated with DG (Figure 1a). Combining both inhibitors (DGO) reduced the puro MFI to levels similar to those of DG alone. For CD4^+^ and CD8^+^ Tconvs in the spleen, the relative puro MFI decreased by 75–80% when glycolysis (DG), OxPhos (O) or both pathways (DGO) were inhibited. In the lungs, the puro MFI of Tregs, CD4^+^ and CD8^+^ Tconvs was drastically diminished in the presence of O and DGO, at the level of control cells not treated with puromycin (C−). By contrast, DG had little or no effect, with some values below or above the median value of 100 (Figure 1a). Similar results were observed when the data were represented in absolute values of puro MFI (Supplementary figure 1). We then deduced glucose and OxPhos dependencies from puro MFI. In the spleen, T‐cell subsets were highly dependent on glucose and OxPhos to generate energy, although Tregs primarily relied on glucose and, to a lesser extent, on OxPhos (Figure 1b). By contrast, in the lungs, Tregs, CD4^+^ and CD8^+^ Tconvs relied mostly on OxPhos rather than glucose to generate energy.

### Higher incorporation of puromycin in dividing T cells *ex vivo* and *in vivo*

To overcome the aforementioned limitations of studying cellular metabolism *ex vivo*, SCENITH was adapted to capture the metabolism of T cells *in vivo*. To this end, we first checked that puromycin administered 1 h before euthanasia could properly be incorporated into T cells. Cells from the spleen and lungs were rapidly dissociated and stained to measure puromycin incorporation. We observed that puromycin was indeed incorporated into Tregs and Tconvs in both tissues, at levels similar to those found *ex vivo* (Supplementary figure 2). As proliferating T cells have a higher metabolic activity than quiescent cells,^8^, ^16^, ^17^ we examined the metabolic activity of T‐cell subsets according to their division state, based on the expression of the nuclear factor Ki67, which is a marker of dividing cells. We indeed observed that Ki67^+^ cells expressed a higher level of puromycin compared with Ki67^−^ cells in Tregs and Tconvs of the spleen and lungs, except for lung CD8^+^ Tconvs (Supplementary figure 2). As a control, we performed similar analyses in the *ex vivo* SCENITH assay. Again, the Ki67^+^ populations had a higher level of puromycin incorporation than the Ki67^−^ populations (Supplementary figure 3a, b). Interestingly, the impact of the DG, O or DGO inhibitors on relative puro MFI was relatively comparable in Ki67^+^ and Ki67^−^ cells, suggesting that quiescent and proliferating T cells had a similar dependence on glucose and OxPhos (Supplementary figure 3c). These data show that puromycin was adequately incorporated into T‐cell subsets *in vivo*.

### The Treg pool is impacted by puromycin and metabolic inhibitors *ex vivo* but not *in vivo*

**Figure 2:**
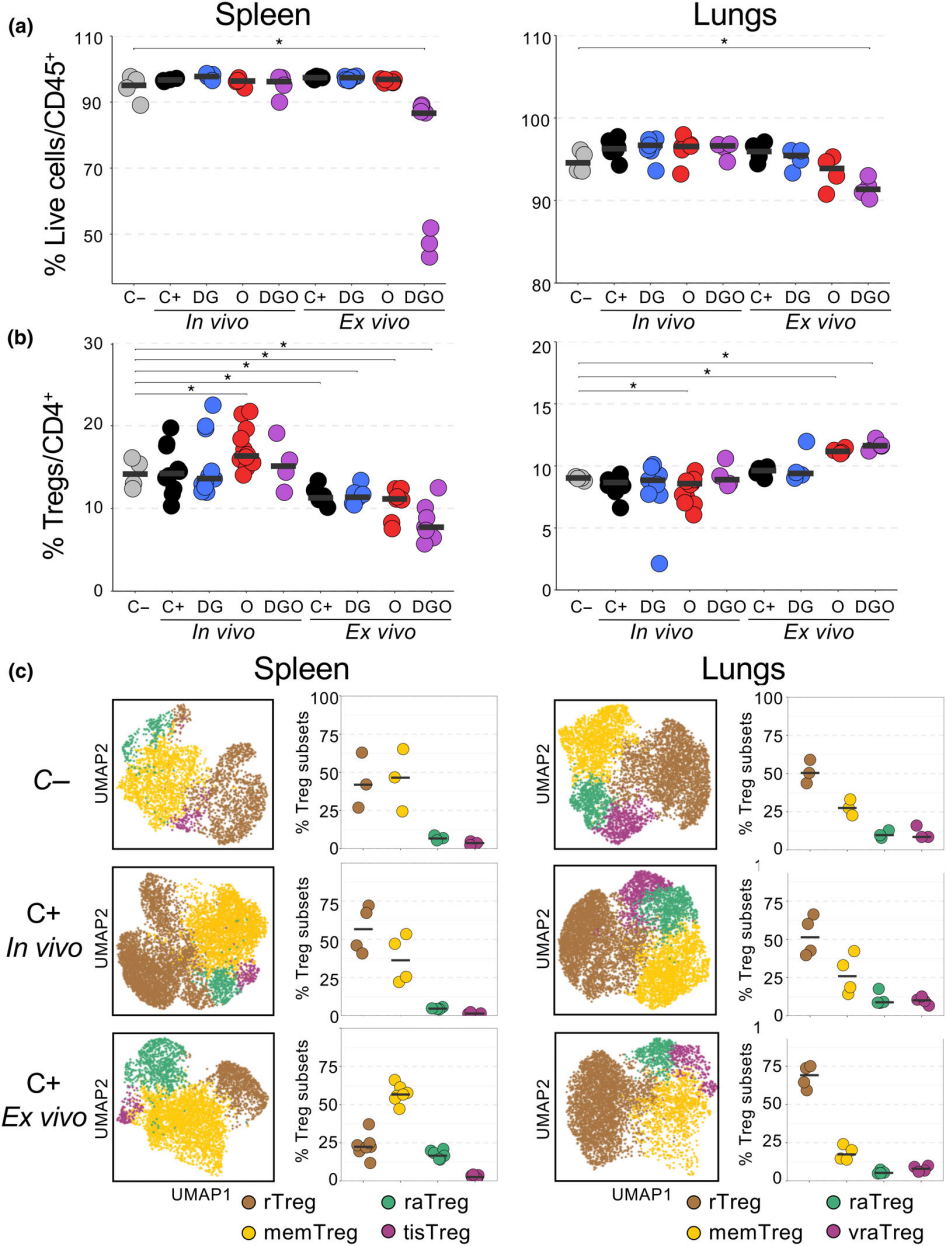
The regulatory T cell (Treg) pool is impacted by puromycin and metabolic inhibitors *ex vivo* but not *in vivo*. **(a, b)** Proportions of splenic and lung viable CD45^+^ cells (a) and Tregs (b) from untreated mice (C–), mice treated *in vivo* with metabolic inhibitors as in Figure 3 or cells treated *ex vivo* with metabolic inhibitors as in Figure 1. Data were compared with the C– control cells. **(c)** Spleen and lung Tregs subsets from untreated mice (C−), mice treated with puromycin *in vivo* (C+, *in vivo*) or cells treated with puromycin *ex vivo* (C+, *ex vivo*). Cells were labeled with a mix of 26 monoclonal antibodies (mAbs) to identify subsets of resting (rTregs), memory (memTregs), recently activated (raTregs) and precursors of nonlymphoid tissue (tisTregs) Tregs in the spleen and subsets of rTregs, memTregs, raTregs and very recently activated Tregs (vraTregs) in the lungs. Uniform manifold approximation and projection (UMAP) showing Treg subsets from a pool of 3 (C–) or 4 mice (C+ *in vivo* and C+ *ex vivo*; left panels) and proportions of the four Treg subsets (right panels). Each symbol represents individual mice and bars represent medians. Number of mice per group: 7–12 mice per condition were used in most conditions (except 4 mice for *ex vivo* lung, *in vivo* in **c** and *in vivo* DGO in **b** conditions). Data are from at least three independent experiments. Statistical analysis was calculated using a Wilcoxon rank‐sum test. *P < 0.05.

Other quality control experiments included the assessment of the impact of puromycin and metabolic inhibitors on the viability of T‐cell subsets. For the *in vivo* SCENITH, mice were treated with DG and O at doses reported in the literature^18^, ^19^: 17 and 2 h before euthanasia and with puromycin 1 h before euthanasia. Compared with CD45^+^ immune cells from untreated control mice (C–), cell viability was significantly affected using *ex vivo* SCENITH for the DGO condition. The same drug combination administered *in vivo* had no effects on the viability of CD45^+^ cells (Figure 2a and Supplementary figure 4). We then assessed the effect of these drugs on Tregs. The normal proportion of Tregs among CD4^+^ T cells was about 15% in the spleen and 10% in the lungs. When drugs were administered *in vivo*, the percentages of Tregs were not or marginally modified (Figure 2b). The same drugs used *ex vivo* had more impact, specifically for the O and DGO conditions, with decreased and increased Treg proportion in the spleen and lungs, respectively (Figure 2b). We then studied Treg subsets using a mix of 26 mAbs by multiparametric flow cytometry. Differential expressions of CD62L, Bcl2, Tcf1, CD44, CTLA4, Ki67, KLRG1, GATA3 and CD25 characterized five Treg populations (Supplementary figure 5 and Supplementary table 1). Resting Tregs (rTregs) were naïve T cells characterized by high CD62L and Bcl2 expression and low expression of activation markers (CD44 and CTLA4). Memory Tregs (memTregs), defined by an increased expression of activation markers (CD44 and CTLA4) in the absence of ongoing proliferation (Ki67^−^ cells), were T cells that were activated and then returned to a resting state. Recently activated (raTregs) and very recently activated Tregs were defined by the expression of markers of activation and proliferation (CD44, CTLA4 and Ki67). CD62L allowed to discriminate these two subsets because cells that have been activated very recently have not yet downregulated this marker. Finally, precursors of nonlymphoid tissue Tregs were defined by KLRG1 expression, a marker of nonlymphoid tissue Tregs (Supplementary figure 5 and Supplementary table 1). Similar Treg populations were previously observed in the spleen and lungs.^20^, ^21^ rTregs, memTregs and raTregs Tregs were present in both the spleen and lungs, whereas tissue Tregs were only identified in the spleen and very recently activated Tregs in the lungs (Figure 2c and Supplementary figure 5). These populations were identified in the *in vivo* and *ex vivo* SCENITH assays but with varying proportions (Figure 2c). In the spleen of control untreated mice (C–), rTregs and memTregs constituted each approximately half of the Treg pool while raTregs and tissue Tregs were minor populations (Figure 2c). In the *in vivo* assay, the proportions of splenic Treg subsets remained broadly unchanged when puromycin was administered compared with untreated mice. By contrast, when splenic Tregs were cultured with puromycin (C+ *ex vivo*), the Treg pool was altered, with increased percentages of memTregs and raTregs and decreased percentages of rTregs (Figure 2c). In the lungs of control untreated mice (C–), the Treg pool comprised 50% rTregs, 25% memTregs and 10% of raTregs and very recently activated Tregs. These proportions were maintained when puromycin was administered *in vivo* but not *ex vivo* as the proportion of rTregs increased while the proportion of memTregs and raTregs decreased (Figure 2c). Overall, these results indicate that puromycin and metabolic inhibitors impacted the proportions of Tregs and Treg subsets in the *ex vivo* but not the *in vivo* SCENITH.

### Under physiological conditions, lung Tregs and Tconvs have an OxPhos‐based metabolism

**Figure 3:**
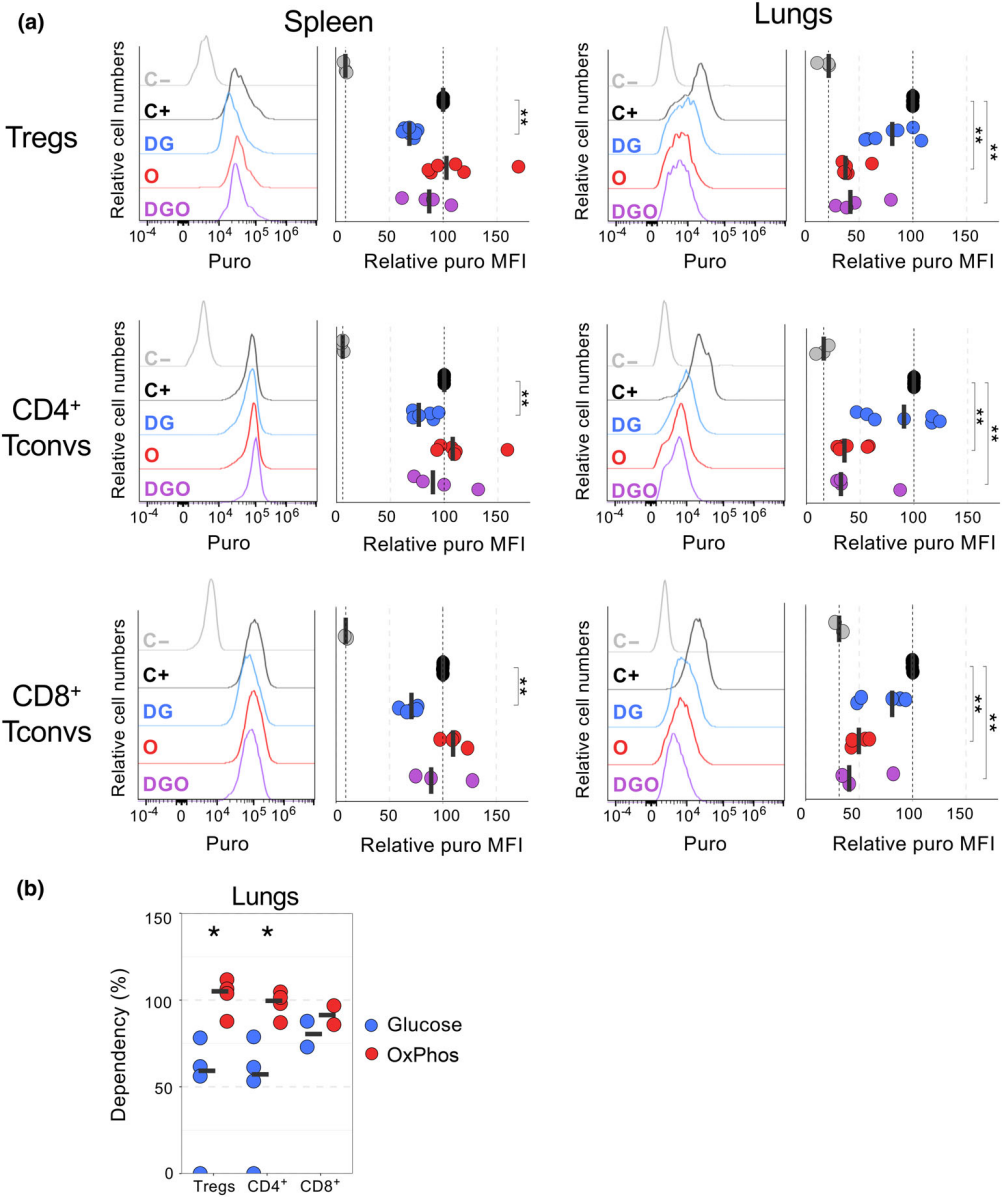
Lung T cells have an oxidative phosphorylation (OxPhos)–based metabolism at a steady state. SCENITH was performed on spleen and lung cells in mice that were injected intraperitoneally with DG, O or both inhibitors 17 and 2 h before euthanasia. Puromycin (puro) was injected intraperitoneally 1 before euthanasia and its incorporation in regulatory T cells (Tregs), CD4^+^ conventional T cells (Tconvs) and CD8^+^ Tconvs was analyzed by flow cytometry. Mice were injected with diluents (C−), puro alone (C+), puro and DG, puro and oligomycin (O) or puro, DG and oligomycin (DGO). **(a)** Representative puro staining (left panels) and puro mean fluorescent intensity (MFI) relative to the puro MFI of C+ control (right panels). **(b)** Glycolysis and OxPhos dependencies were calculated as in Figure 1. Each symbol represents individual mice and bars represent medians. Data are from at least three independent experiments, with 5–7 mice per group in all conditions (except 3 for DGO) **(a)** and 2–4 mice per group **(b)**. Statistical analysis was calculated using a Wilcoxon rank‐sum test. *P ≤ 0.05 and **P ≤ 0.01.

To study cell metabolism *in vivo*, we measured the impact of DG and O administration on puromycin incorporation. In the spleen, DG administration significantly but only partially reduced puromycin incorporation in Tregs and Tconvs, compared with control mice not treated with puromycin (C−; Figure 3a). Surprisingly, injection of O appeared to have no effect, and coinjecting DG and O (DGO) only had a minimal impact on puro MFI (Figure 3a). These results may suggest that these inhibitors had no or moderate effect on T‐cell metabolism in the spleen. By contrast, in the lungs, when O or DGO were administered, puro MFI was significantly decreased in Tregs and Tconvs, at levels reaching the C– control (background fluorescence; Figure 3a). No significant differences in puromycin incorporation were noted in mice treated with DG. The analysis of absolute puro MFI yielded similar trends (as relative puro MFI) but was not statistically different (except for lung CD8^+^ Tconvs) because of interexperimental variabilities (Supplementary figure 6). The effect of DG and O on puromycin incorporation was similar in proliferating (Ki67^+^) and quiescent (Ki67^−^) T cells (Supplementary figure 7). Glucose and OxPhos dependencies could be calculated for T cells in the lungs but not in the spleen, as puro MFI levels in the DGO condition were in the range of the C+ control. In the lungs, Tregs and Tconvs were highly dependent on OxPhos (dependency close to 100%) and partially on glucose, with dependency of 55% for Tregs and CD4^+^ Tconvs and 80% for CD8^+^ Tconvs (Figure 3b). In the lungs, T cells are thus highly dependent on OxPhos to produce energy at a steady state. We finally assessed the metabolism of T cells in the inflamed context of a lymph node draining an implanted tumor. In a preliminary experiment, we observed a partial dependence of T‐cell metabolism on glucose rather than OxPhos (Supplementary figure 8). These data demonstrate the feasibility of implementing the SCENITH experimental procedure to study the metabolism of T cells *in vivo*.

### Foxp3 expression is affected by the metabolic activity of Tregs

**Figure 4:**
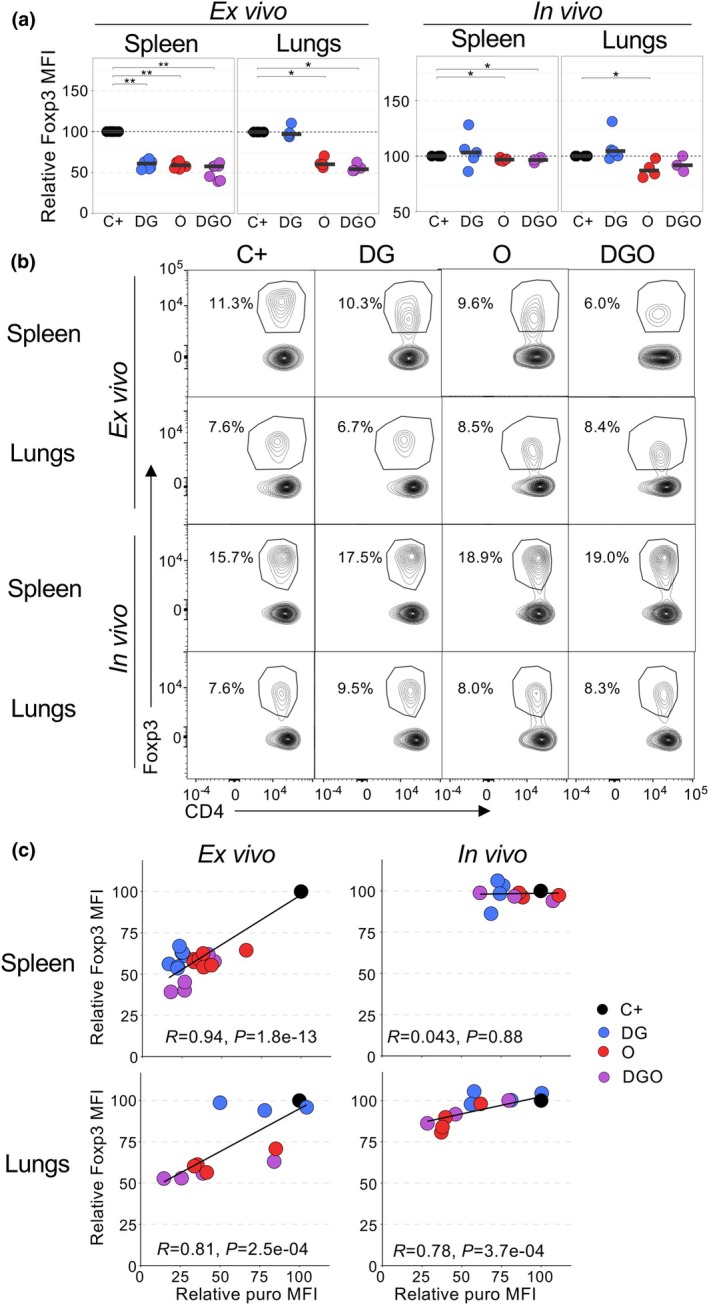
Glycolysis and oxidative phosphorylation (OxPhos) inhibitors reduce Foxp3 expression of regulatory T cells (Tregs) *ex vivo*. Foxp3 expression in spleen and lung Tregs from mice treated *in vivo* with metabolic inhibitors as in Figure 3 or cells treated *ex vivo* with metabolic inhibitors as in Figure 1. **(a)** Mean fluorescent intensity (MFI) of Foxp3 expression relative to the puromycin (puro) MFI of C+ control on gated Foxp3^+^ cells. **(b)** Representative Foxp3 expression on gated CD4^+^ cells. **(c)** Correlation plots displaying the relative Foxp3 MFI versus the relative puro MFI on gated Foxp3^+^ cells. Each symbol represents individual mice and horizontal bars represent medians. Data are from at least two independent experiments (4–7 mice per group). Statistical analysis was calculated using **(a)** a Wilcoxon rank‐sum test and **(c)** a Pearson correlation coefficient test.*P ≤ 0.05. memTreg, memory Treg; rTreg, resting Treg; raTreg, recently activated Treg; tisTreg, tissue Treg; UMAP, uniform manifold approximation and projection.

Finally, we assessed whether *in vitro* and *in vivo* inhibition of glycolysis and OxPhos could impact the expression of activation and Treg markers. Foxp3 is a transcription factor that plays a critical role in the identity and suppressive function of Tregs.^22^, ^23^, ^24^ Maintaining a high level of Foxp3 expression is essential in Treg biology. We therefore evaluated if DG and O could affect its expression. In *ex vivo* SCENITH, Foxp3 expression was significantly decreased across all conditions for splenic Tregs and in the O and DGO conditions for lung Tregs (Figure 4a, b and Supplementary figure 9). In *in vivo* SCENITH, DG had no impact, while O and DGO induced only a minor diminution of Foxp3 expression for splenic and lung Tregs (Figure 4a, b and Supplementary figure 9). Interestingly, when Foxp3 expression was clearly affected by these metabolic inhibitors (in *ex vivo* conditions and *in vivo* for lung Tregs), Foxp3 expression positively corelated with puromycin expression (Figure 4c), suggesting that Foxp3 expression was dependent on the metabolic activity of Tregs. We then analyzed other transcription factors. Interestingly, expression of GATA3, TCF1 and RORγt decreased under certain conditions, a phenomenon that was generally more visible in *ex vivo* SCENITH than in *in vivo* SCENITH. For example, *ex vivo* treatment with DG and O resulted in reduced GATA3 expression on splenic and pulmonary Tregs (Supplementary figure 10). The same was observed with the O condition for TCF1. For most of the other studied markers, DG, O and DGO had minimal or variable effects precluding any clear conclusion (Supplementary figures 11 and 12). Taken together, these results indicate that the metabolic activity of Tregs is important for maintaining Foxp3 expression.

## DISCUSSION

This study presents two key findings. Methodologically, we demonstrate the proof of principle that the metabolism of rare cells can be studied *in vivo*. Biologically, we unveil that lung Tregs and Tconvs rely on OxPhos, rather than glycolysis, to maintain basal energy production.

We provide the evidence of principle that the SCENITH assay can be adapted to assess the metabolic profile of cells *in vivo*. This is possible because puromycin largely diffuses in different tissues after administration, as shown previously,^25^, ^26^ and that its incorporation into proteins can be detected by flow cytometry with a specific mAb. Further, several metabolic inhibitors diffuse systemically and rapidly after administration and could be detected in multiple tissues, including the spleen, colon,^18^, ^19^ lymph nodes^27^ and tumors.^28^, ^29^ A possible limitation of our *in vivo* SCENITH assay would be that some metabolic inhibitors insufficiently diffuse in specific tissues. We observed that co‐injected DG and O strongly inhibited puromycin incorporation in lung T cells, illustrating that these inhibitors efficiently penetrated the tissue. However, these inhibitors had minimal action on spleen T cells, which can be interpreted in two ways. Either DG or O insufficiently diffuses in the spleen, which is possible even though half the dose we used here had an effect in previous studies on this organ.^30^, ^31^ We could not inject a higher dose of the metabolic inhibitors because of their high *in vivo* toxicity. In future studies, we may consider using molecular engineering drugs derived from metabolic inhibitors to reach higher doses locally in a given tissue. We also tested the effect of injecting DG and O only once at the same dose (2 h before euthanizing) instead of injecting the inhibitors twice. In both the spleen and lungs, one injection had no impact on T‐cell metabolic activity (Supplementary figure 13), explaining why we used two injections throughout our study. Alternatively, spleen T cells may not be affected by these drugs over the few hours of the experiment because of their low basal metabolism at a steady state, which may not be observed in the *ex vivo* SCENITH assay because cellular stress may alter their metabolism (see below).

The *in vivo* SCENITH assay has remarkable potential and applications. Detection of puromycin can be combined with a mix of 20–40 mAbs to identify various cell subsets and activation markers, as we did in the present study. The metabolic profile of different lymphoid and myeloid populations can consequently be determined simultaneously in the same sample. Besides, inhibitors other than O and DG have been used *in vivo* to block major metabolic pathways, such as dimethyl malonate to block the tricarboxylic acid cycle,^32^ etomoxir to block fatty acid oxidation,^33^ 5‐(tetradecyloxy)‐2‐furoic acid to block fatty acid synthase^34^ or 6‐diazo‐5‐oxo‐l‐norleucine to block glutaminolysis.^35^ These different metabolic pathways could also be possibly evaluated on rare cells *in vivo* after implementing an improved version of the *in vivo* SCENITH assay in the future. We should point out that DG not only blocks glycolysis but also N‐glycosylation, suggesting that more specific inhibitors would be preferable in future studies.

The *in vivo* SCENITH assay should more accurately assess the cell metabolic activity in their natural environment than *ex vivo* assays (classical SCENITH or SeaHorse). Indeed, the stress caused by extracting cells from their natural tissue niche followed by their culture may induce cellular toxicity and metabolic modifications. We observed that combining 2‐DG and O in culture affected the viability of CD45^+^ cells but not when these drugs were administered *in vivo*. However, dead cells may be not detected *in vivo* because they have been eliminated rapidly by phagocytic cells. Besides, cell extraction and culture with some inhibitors modified Treg and Treg subset proportions, suggesting that some Treg subsets were particularly sensitive to this sequential experimental procedure. These alterations were not or minimally observed in the *in vivo* assay. For all these reasons, the *in vivo* SCENITH assay has promising potential to study the metabolism of cells *in vivo* accurately.

When studying the cellular metabolism of rare cells, other methods such as RNA sequencing and metabolomics should be considered because of recent improvements. Complex algorithms such as COMPASS^36^ can use single‐cell RNA sequencing data to infer single‐cell metabolic states. Metabolomics techniques have significantly improved, allowing analysis of metabolites of rare cell cells.^37^ One of the drawbacks of these techniques is that cells have to be purified before RNA or metabolite extraction, altering their metabolic profile because of cell extraction followed by hours of purification. Integrating data from all these new methods should yield a more comprehensive understanding of the cell's metabolic profile.

Most studies investigating the metabolism of T cells have been performed *in vitro*. It is well‐described that resting T cells have a low basal activity and predominantly use OxPhos rather than glycolysis. After T cell activation, glycolysis and OxPhos are highly increased to produce abundant biomass, supporting effector functions and proliferation.^1^, ^38^, ^39^ Because of cultural artifacts, some results obtained in these studies may be inaccurate. For example, it is well‐known that pyruvate derived from glycolysis is predominantly transformed into lactate in cultured activated T cells. However, when this question was addressed *in vivo*, it was observed that most of the pyruvate produced by glycolysis fuels the tricarboxylic acid cycle rather than being converted to lactate.^38^ Therefore, results obtained *in vitro* or *ex vivo* should be cautiously interpreted before extrapolating them to physiological conditions.

Studies comparing the metabolism of Tregs and Tconvs *in vitro* led to debated findings. Some studies suggest that Tregs use OxPhos rather than glycolysis,^40^, ^41^, ^42^, ^43^, ^44^, ^45^ and others the opposite.^46^, ^47^ Some of these controversial observations may be due to different *in vitro* conditions. For instance, Tregs, but not Tconvs, were cultured with transforming growth factor‐β in many studies. However, transforming growth factor‐beta inhibits glycolysis and promotes OxPhos, leading to the erroneous conclusion that Tregs use predominantly OxPhos.^48^ Again, these conflicting data highlight the limitations of studying cell metabolism *in vitro* and should be cautiously interpreted. Most reliable data come from studies using mice that have conditional knockout for critical metabolic regulators. Using this approach, it appears that the metabolism of Tregs and Tconvs shares many similarities. Thus, as Tconvs, Tregs require OxPhos to survive and an increase in both glycolysis and OxPhos to divide and acquire effector functions.^2^, ^49^, ^50^, ^51^, ^52^ However, tumor‐infiltrating Tregs and Tconvs appear to have different metabolic states. Contrary to Tconvs, tumor‐infiltrating Tregs can switch a metabolic program, conferring them the capacity to survive and function in a lactate‐rich glucose‐poor microenvironment by efficiently capturing and metabolizing lactate and lipids.^43^, ^53^, ^54^, ^55^, ^56^, ^57^ Tumor‐infiltrating Tregs consequently appear to have a high OxPhos and low glycolysis metabolic activity.

Besides tumor‐infiltrating T cells, very few studies have analyzed the metabolic program of T cells in nonlymphoid tissues at steady state, likely due to the challenges of investigating the cellular metabolism of rare cells. Two studies found that CD8^+^ T cells resident in the skin and intestine have a high ability to capture and oxidize extracellular lipids, fueling the tricarboxylic acid cycle.^58^, ^59^ Furthermore, T‐cell fitness to nonlymphoid tissues requires mitochondrial adaptation to support an OxPhos‐oriented metabolism.^60^, ^61^ These studies indicate that T cells have to polarize their metabolism toward OxPhos to be adapted to nonlymphoid tissue environments, where the metabolite composition landscape is radically different from lymphoid tissues.^62^, ^63^, ^64^

A limited number of studies analyzed the metabolism of freshly purified T cells at a steady state using the *ex vivo* SCENITH assay. For human blood T cells, one suggested that T cells were highly dependent on glucose,^65^ another one on OxPhos^66^ and a third one on both glucose and OxPhos.^67^ In mice, two studies suggested that splenic and lymph node CD4^+^ T cells were OxPhos dependent,^12^, ^68^ one suggested that splenic CD8^+^ T cells were glucose dependent (OxPhos was not analyzed)^10^ and another one suggested that lung CD4^+^ T cells were dependent on both glucose and OxPhos.^13^ These different results could be explained by the fact that different cells were analyzed (antigen‐specific versus bulk T cells), as well as different concentrations of inhibitors and different anti‐puromycin mAbs were used. In any case, the data are too conflicting to draw any conclusions at the moment, except that human blood CD4^+^ and CD8^+^ T cells would have similar metabolic profiles. The *ex vivo* SCENITH will profit from standardization to improve reliability and reproducibility between different laboratories.

The following main conclusions on the metabolism of T cells at steady state can be drawn from our data: (1) CD4^+^ and CD8^+^ Tconvs of the spleen and lungs have similar OxPhos and glucose dependence. These findings were observed in both the *ex vivo* and *in vivo* SCENITH assays and are compatible with previous reports using *ex vivo* SCENITH assessing blood human T cells.^65^, ^66^, ^67^ (2) Tregs and Tconvs have similar metabolic profiles, except for spleen cells analyzed using SCENITH *ex vivo*, where Tregs would be less dependent on OxPhos than Tconvs. Thus, our findings further support that Tregs and Tconvs have globally similar metabolic activity, with the notable exception of tumor‐infiltrating T cells, as discussed above. (3) Splenic Tregs and Tconvs depend on both glycolysis and OxPhos *ex vivo*. This could not be confirmed *in vivo*, possibly as a result of technical limitations or low basal metabolic activity, as previously discussed. Comparing our data with the few published reports using an *ex vivo* SCENITH assay^10^, ^12^, ^68^ is challenging because these studies showed conflicting data, as discussed above, and metabolic profiles of CD4^+^ Tconvs and Tregs were not investigated separately. (4) The metabolism of lung T cells is more reliant on OxPhos than glucose, which was observed both when performing SCENITH *ex vivo* and *in vivo*. This observation is one of the major findings of our work, which strengthens the important concept that T cells in nonlymphoid tissues would adopt an OxPhos‐based rather than glucose‐based metabolism, as discussed above. This metabolic switch may be driven by the low glucose abundance in these tissues, promoting metabolic adaptation to capture and metabolize lactate and lipids. An extended study of T cells in various nonlymphoid tissues using our *in vivo* SCENITH assay should confirm this critical question.

The impact of DG and O on Foxp3 expression in *ex vivo* assays is intriguing. Inhibiting glycolysis and OxPhos in splenic T cells and OxPhos in lung T cells led to a significant loss of Foxp3. Interestingly, Foxp3 loss was correlated with a decrease in puromycin incorporation. The most likely explanation is that Foxp3 maintenance highly depends on a fully functional metabolic activity and protein translation. Altering active metabolic pathways would decrease adenosine triphosphate levels, resulting in a reduction of protein translation, having an immediate impact on proteins that have a very rapid turnover, such as Foxp3.^69^ Other transcription factors, such as GATA3, were similarly impacted by these metabolic inhibitors, which may be because of their rapid turnover. This phenomenon was more easily revealed *ex vivo* than *in vivo*, possibly because of better inhibition of metabolic pathways.

In conclusion, this study presents an innovative technique, adapted from SCENITH, for assessing the metabolism of rare T cells *in vivo*. Specifically, we established that lung‐resident Tregs and Tconvs depend primarily on OxPhos rather than glycolysis for energy production. The *in vivo* SCENITH assay is effective in overcoming the artifacts and stress induced by *ex vivo* manipulations, providing a more accurate representation of cellular metabolic states. This discovery underlines the importance of studying cellular metabolism in natural tissue microenvironments. These results contribute to a better understanding of T‐cell biology and highlight the potential of SCENITH *in vivo* to elucidate metabolic adaptations in different tissue environments.

## METHODS

### Mice

C57BL/6 mice, obtained from Janvier Labs (Le Genest‐Saint‐Isle, France), were housed under specific pathogen‐free conditions. Experiments were performed with male and female mice of 6–11 weeks of age. The experimental protocol was approved by the French Department of Research and higher Education under the numbers 26677‐202007211707430 and 39799‐2022120217463495 in compliance with European Union guidelines.

### *In vivo* treatment

Mice were injected intraperitoneally with 1 g kg^−1^ DG (reference D6134, Merck KGaA, Darmstadt, Germany) or 0.25 mg kg^−1^ oligomycin A (reference 75351; Merck KGaA) or both inhibitors 17 and 2 h before euthanasia. Puromycin (50 mg kg^−1^; reference P7255; Merck KGaA) was then injected intraperitoneally 1 h before euthanasia.

### Tissue digestion for flow cytometry analysis

After euthanasia, mice were perfused by an intracardiac injection of cold phosphate‐buffered saline. The spleen was mechanically dilacerated on a 70‐μm cell strainer. Red blood cells were lysed with ammonium chloride for 1 min and washed. The lung tissue was cut into small pieces and digested enzymatically after undergoing SCENITH with the mouse Lung Dissociation Kit (reference 130‐095‐927; Miltenyi Biotec, Bergisch Gladbach, Germany) in 12‐well plates, for 40 min at 37°C with a shaking of 120 rounds per min. Digested lung tissue was then filtered on a 70‐μm cell strainer.

### *Ex vivo* treatment

Splenic cell suspensions were seeded in a 96‐well plate at 1 × 10^6^ cells mL^−1^ and cultured with Roswell Park Memorial Institute (RPMI) 1640 (reference 11875093, Thermo Fisher Scientific, Waltham, MA, USA) supplemented with 10% fetal calf serum, 2 mM l‐glutamine, 10 mM HEPES [4‐(2‐hydroxyethyl)‐1‐piperazineethanesulfonic acid] and 50 μM beta‐mercaptoethanol for 50 min at 37°C with a shaking of 120 rounds per min. Cells were treated with either 100 mM DG (reference D6134; Merck KGaA) or 1 μM of O (reference 75 351; Merck KGaA) or both inhibitors. About 10 μg mL^−1^ puromycin (reference A1113803; Thermo Fisher Scientific) was added for the last 20 min of the culture. The incubation was stopped by adding cold phosphate‐buffered saline, cells were washed and then prepared for flow cytometry analysis. Retrieved lung tissues were cut into small pieces and seeded in a 12‐well plate. Sliced lung tissues were then treated with either 100 mM DG (reference D6134, Merck KGaA) or 1 μM O (reference 75351, Merck KGaA) or both inhibitors in RPMI 1640 (reference 11875093, Thermo Fisher Scientific) supplemented with 10% fetal calf serum, 2 mM l‐glutamine, 10 mM HEPES and 50 μM beta‐mercaptoethanol for 50 min at 37°C, with a shaking of 120 rounds per min. After 30 min, 10 μg mL^−1^ puromycin (reference P7255, Merck KGaA) was added for the remaining last 20 min of the culture. Lung tissues were then digested as described above and prepared for flow cytometry analysis.

### Antibody staining for flow cytometry

Single‐cell suspensions were transferred in U‐bottom 96‐well plates for antibody staining. Cell survival was assessed with fixable viability dyes (LIVE/DEAD Blue (reference L34961, Thermo Fisher Scientific) or Fixable Viability Dye eFluor780 (reference 65‐0865‐14, Thermo Fisher Scientific). Nonspecific antibody binding to Fc receptors was blocked using the 2.4G2 mAb. Cells were first incubated with mAbs specific for molecules expressed at the cell membrane. Then, cells were incubated for 60 min at 4°C with the Foxp3/Transcription Factor Staining Buffer (reference 00‐5521‐00, Thermo Fisher Scientific) to fix and permeabilize the cells following the manufacturer's protocol. Intracellular staining was performed at 4°C for 45 min in a permeabilization buffer (reference 00‐5521‐00, Thermo Fisher Scientific). All antibodies used in this study are listed in Table 1.

**Table 1:** Antibodies used for flow cytometry.

Antigen	Fluorophore	Clone	Producer	Catalog No.
Bcl2	PE‐Cy7	C10‐3	Thermo Fisher Scientific	25‐6992‐42
CCR2	BUV661	475 301	BD Biosciences, San Diego, CA, USA	750042
CD25	BV605	PC61	BioLegend, San Diego, CA, USA	102036
CD3	BUV563	145‐2C11	BD Biosciences	749277
CD3	BUV737	145‐2C11	BD Biosciences	612771
CD4	BUV496	GK1.5	BD Biosciences	612952
CD44	PerCP	IM7	BioLegend	103035
CD44	PerCP‐Cy5.5	IM7	Thermo Fisher Scientific	45‐0441‐82
CD45	BUV395	30‐F11	BD Biosciences	564279
CD62L	BV650	MEL‐14	BD Biosciences	564108
CD73	PerCP‐Cy5.5	TY/11.8	BioLegend	127213
CD8a	BUV805	53‐6.7	BD Biosciences	564920
c‐MAF	eFluor 450	symOF1	Thermo Fisher Scientific	48‐9855‐42
CTLA‐4	PE‐CF594	UC10‐4F10‐11	BD Biosciences	564332
Eomes	PerCP‐eF710	Dan11mag	Thermo Fisher Scientific	46‐4875‐82
Foxp3	FITC	FJK‐16s	Thermo Fisher Scientific	11‐5773‐82
Foxp3	eFluor 450	FJK‐16s	Thermo Fisher Scientific	48‐5773‐82
Gata‐3	PE‐Cy5	TWAJ	Thermo Fisher Scientific	15‐9966‐41
Granzyme B	APC‐Fire750	QA16A02	BioLegend	372210
ICOS	BV711	7E.17G9	BD Biosciences	740763
Ki67	AF532	SolA15	Thermo Fisher Scientific	58‐5698‐82
Ki67	PE	SolA15	Thermo Fisher Scientific	12‐5698‐82
KLRG1	BUV615	2F1	BD Biosciences	751191
KLRG1	BV510	2F1	BioLegend	138421
PD1	BUV737	j43	BD Biosciences	749422
PD1	Super Bright 600	j43	Thermo Fisher Scientific	63‐9985‐82
Puromycin	AF488	12D10	Merck KGaA	MABE343‐AF488
Puromycin	AF647	12D10	Merck KGaA	MABE343‐AF647
NRP1	BV750	V46‐1954	BD Biosciences	752452
RORγt	BV421	Q31‐378	BD Biosciences	562894
ST2	BV480	U29‐93	BD Biosciences	746701
T‐bet	BV786	O4‐46	BD Biosciences	564141
TCF‐7/TCF‐1	R718	S33‐966	BD Biosciences	567587

### Flow cytometry analysis and statistics

Cells were acquired on an LSRII (BD Bioscience, San Diego, CA, USA) or an Aurora spectral (Cytek Biosciences, Fremont, CA, USA) cytometer. Flow cytometry data were analyzed using FlowJo software (BD Bioscience, version 10.8.2) and OMIQ (version 22.1, Dotmatics, Boston, MA, USA). Uniform manifold approximation and projections were produced using OMIQ. Plots were produced with ggplot2 (version 3.5.0) and statistical analyses were performed using the packages ggpubr (version 0.6.0) and rstatix (version 0.7.2) in R (version 4.3.1; R Foundation, Vienna, Austria). *P*‐values are indicated as * for *P* ≤ 0.05, ** for *P* ≤ 0.01, *** for *P* ≤ 0.001 and **** for *P* ≤ 0.0001.

### MCA205 tumor model

Mice were subcutaneously injected in the flank with 0.2 million MCA205 fibrosarcoma cells. *In vivo* treatment with metabolic inhibitors (DG, O and DGO), followed by puromycin, was administered as previously described on days 12 and 13 post‐tumor inoculation. After euthanasia, draining lymph nodes were collected, processed and stained for flow cytometry analysis.

## AUTHOR CONTRIBUTIONS

**Aristeidis Roubanis:** Conceptualization; data curation; formal analysis; methodology; writing – original draft; writing ‐ review and editing. **Morgane Hilaire:** Data curation; formal analysis; methodology. **Morgane Le Teuff:** Data curation; formal analysis; methodology. **Odile Devergne:** Data curation; investigation. **Tim Sparwasser:** Data curation; investigation. **Luciana Berod:** Data curation; formal analysis. **Benoît L Salomon:** Conceptualization; data curation; formal analysis; funding acquisition; investigation; supervision; writing – original draft; writing – review and editing.

## CONFLICT OF INTEREST

The authors do not have any conflict of interest.

## Supporting information


**Supplementary figures 1**.
**Supplementary figures 2**.
**Supplementary figures 3**.
**Supplementary figures 4**.
**Supplementary figures 5**.
**Supplementary figures 6**.
**Supplementary figures 7**.
**Supplementary figures 8**.
**Supplementary figures 9**.
**Supplementary figures 10**.
**Supplementary figures 11**.
**Supplementary figures 12**.
**Supplementary figures 13**.
**Supplementary table 1**.

## Data Availability

The data that support the findings of this study are available from the corresponding author upon reasonable request.
