# Supplementary material for: A new method to measure cell metabolism of rare cells in vivo reveals a high oxidative phosphorylation dependence of lung T cells
Source: Immunol Cell Biol. 2025 Apr 23;103(7):600–14. doi: 10.1111/imcb.70018 (PMC12392706; doi:10.1111/imcb.70018)
Supplement: Supplementary file 1 — Supplementary figures 1. Supplementary figures 2. Supplementary figures 3. Supplementary figures 4. Supplementary figures 5. Supplementary figures 6. Supplementary figures 7. Supplementary figures 8. Supplementary figures 9. Supplementary figures 10. Supplementary figures 11. Supplementary figures 12. Supplementary figures 13. Supplementary table 1. [file IMCB-103-600-s001.pdf]

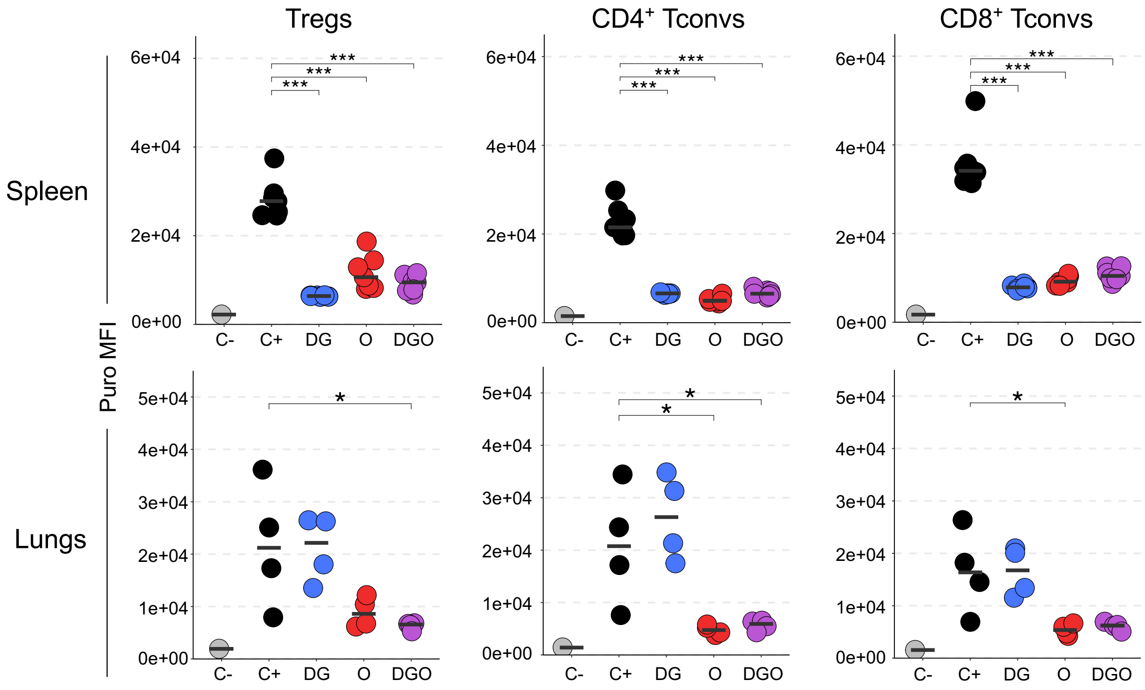

Supplementary figure 1. Puro MFI of T cells from *ex vivo* SCENITH^{TM} experiments. Puro MFI of spleen and lung T cells that underwent SCENITH^{TM} *ex vivo*, processed as in figure 1. Each symbol represents individual mice and bars are medians. Data are from at least three independent experiments (7 mice per group for the spleen and 4 mice per group for the lungs). Statistical analysis was performed using a Wilcoxon rank sum test.

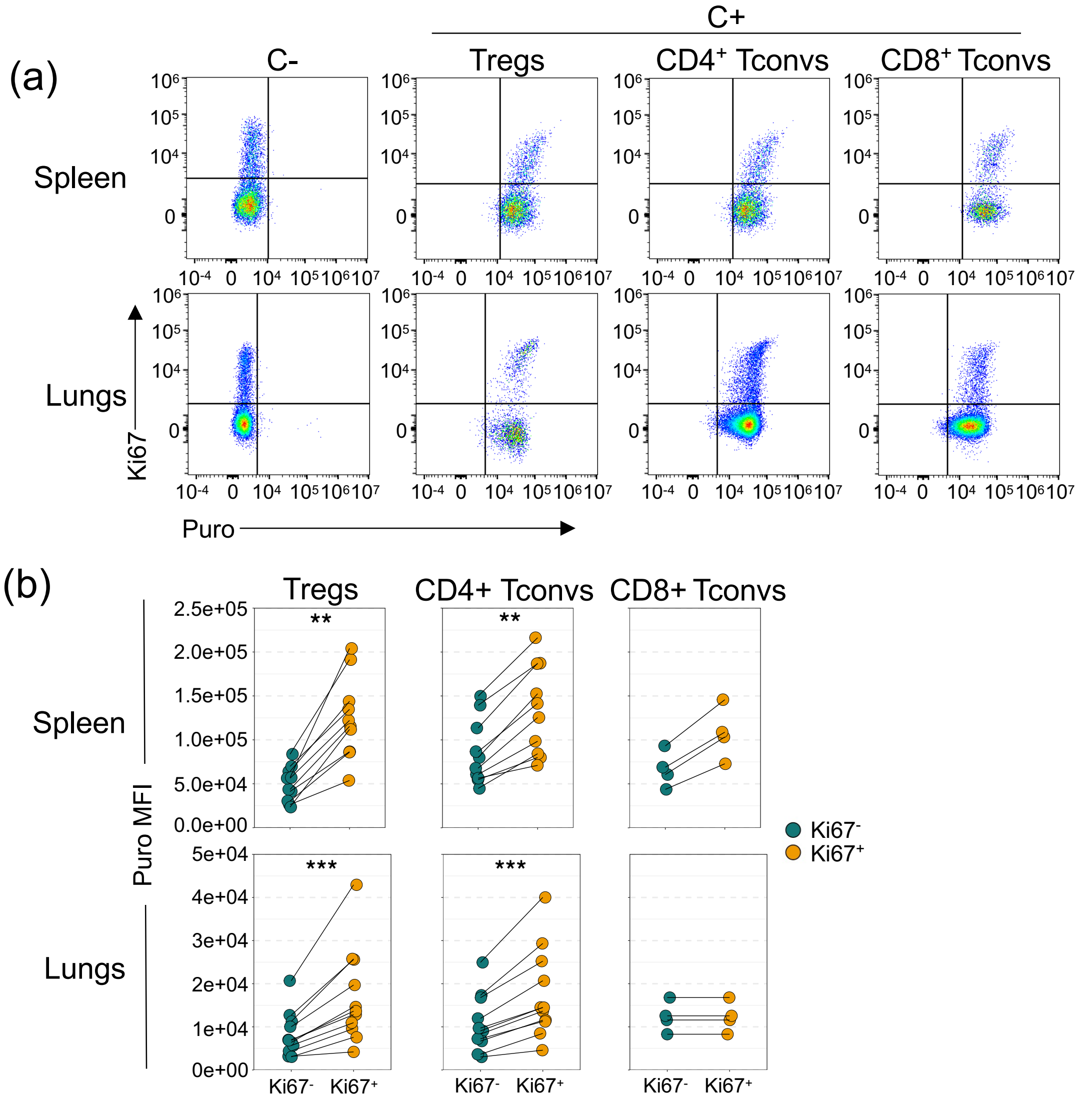

Supplementary figure 2. Increased puromycin incorporation in dividing T cells following *in vivo* administration. Puromycin incorporation assessed by flow cytometry in spleen and lung T cells (Tregs, CD4^{+} and CD8^{+} Tconvs) of mice treated the drug one hour before euthanasia (C+) and untreated mice (C-, gated on CD4^{+} cells). (a) Representative dot plot showing puromycin (puro) vs Ki67 staining in Tconvs and Tregs. (b) Comparison of puro MFI between Ki67^{-} and Ki67^{+} T cells for each individual mouse from at least three independent experiments (11 mice per group for Tregs and CD4^{+} Tconvs and 4 mice per group for CD8^{+} Tconvs). Statistical analysis was performed using a Wilcoxon signed-rank test.

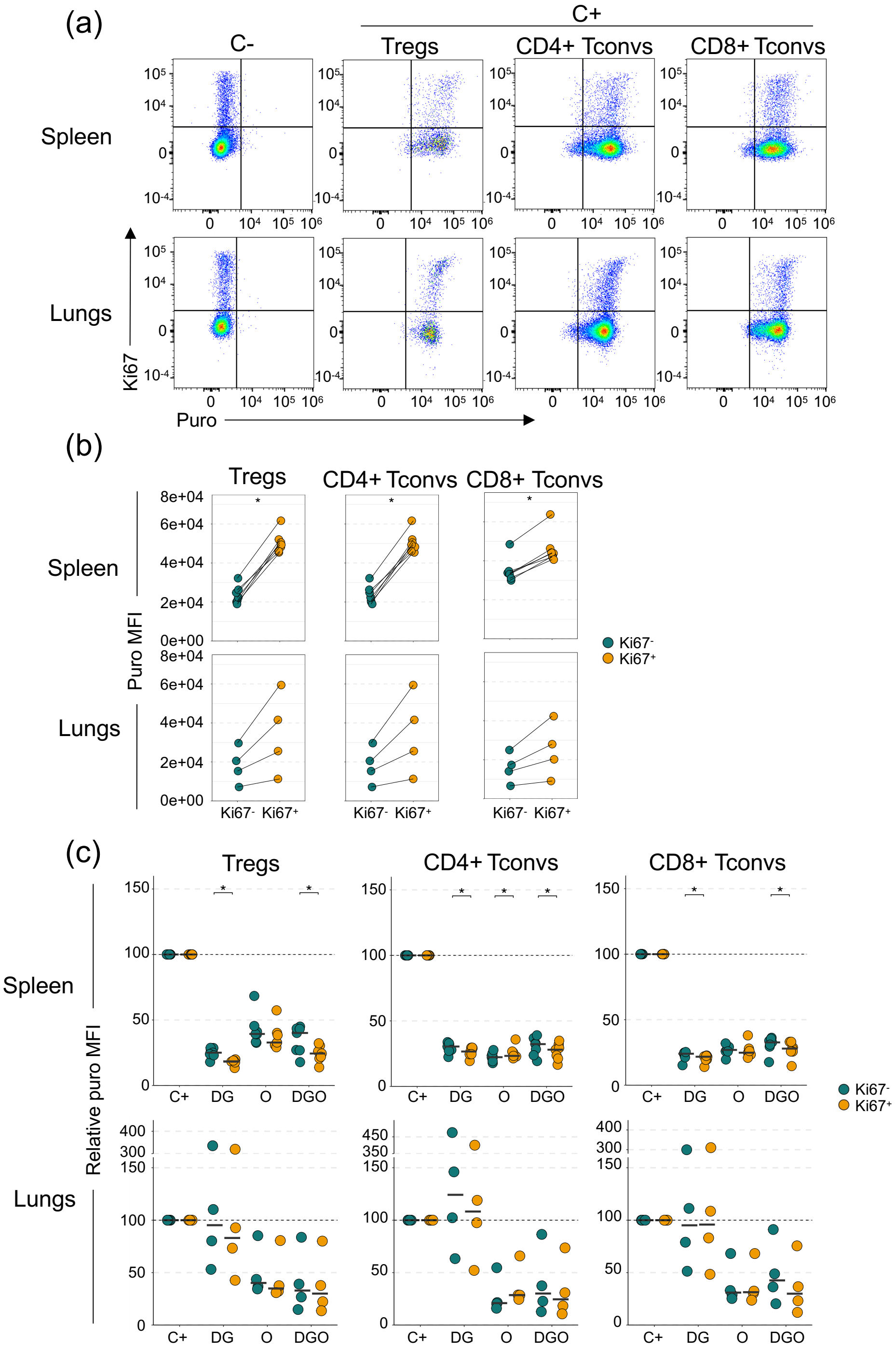

Supplementary figure 3. Puromycin staining of T cells based on cell division from *ex vivo* SCENITH^{TM} experiments. (a, b) Spleen and lung cells were cultured with (C+) or without (C-, gated on CD4$^+$ cells) puromycin, as in figure 1 and analyzed by flow cytometry. (a) Representative dot plot showing puromycin (puro) vs Ki67 staining in Tregs and Tconvs. (b) Comparison of puro MFI between Ki67$^-$ and Ki67$^+$ Tregs and Tconvs. (c) Spleen and lung cells were cultured with metabolic inhibitors (puromycin, 2-DG, oligomycin) as in figure 1 and analyzed by flow cytometry. Comparison of relative puro MFI between Ki67$^-$ and Ki67$^+$ in Tregs and Tconvs. Each symbol represents individual mice and bars represent medians from at least three independent experiments (11 mice per group for Tregs and CD4$^+$ Tconvs and 4 mice per group for CD8$^+$ Tconvs). Statistical analysis was performed using a Wilcoxon signed-rank test.

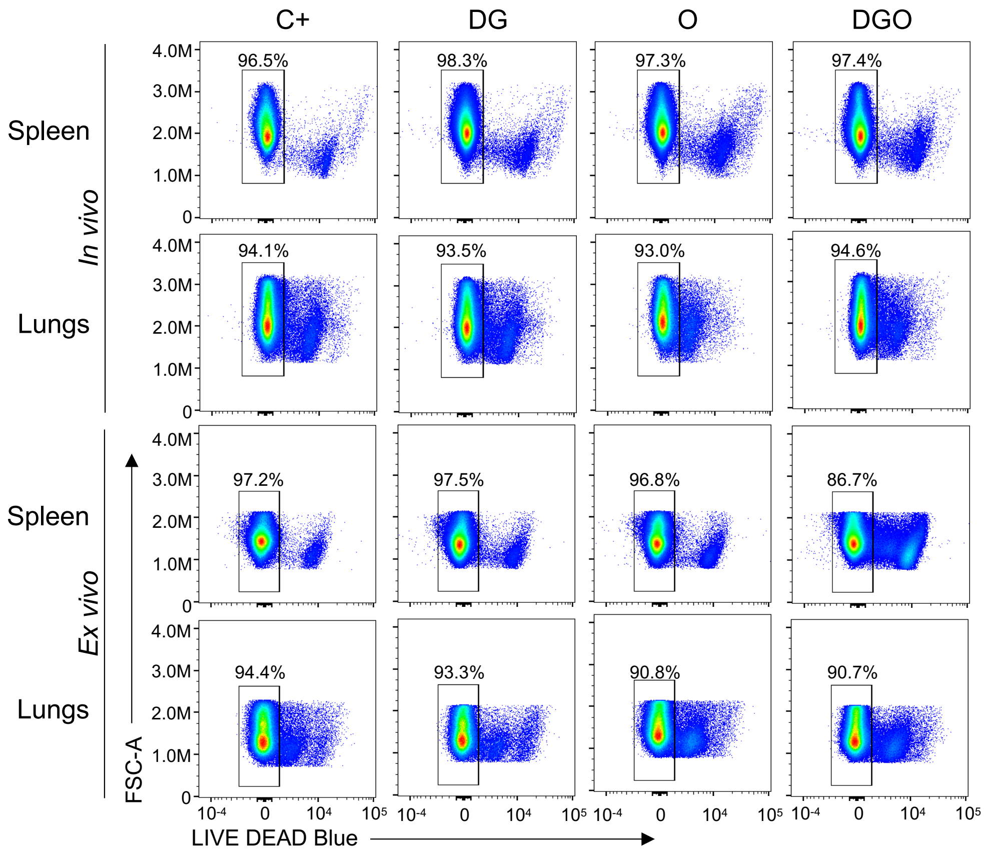

Supplementary figure 4. *In vivo* and *ex vivo* effect of metabolic inhibitors on the viability of CD45$^+$ cells. Representative flow cytometry dot plots on gated CD45$^+$ cells showing forward side scatter versus LIVE DEAD Blue dye in splenic and lung cells from mice treated *in vivo* with metabolic inhibitors as in figure 3 or cells treated *ex vivo* with metabolic modulators as in figure 1. The percentages of viable CD45$^+$ cells are displayed. Data are representative of at least three independent experiments (4-7 mice per group).

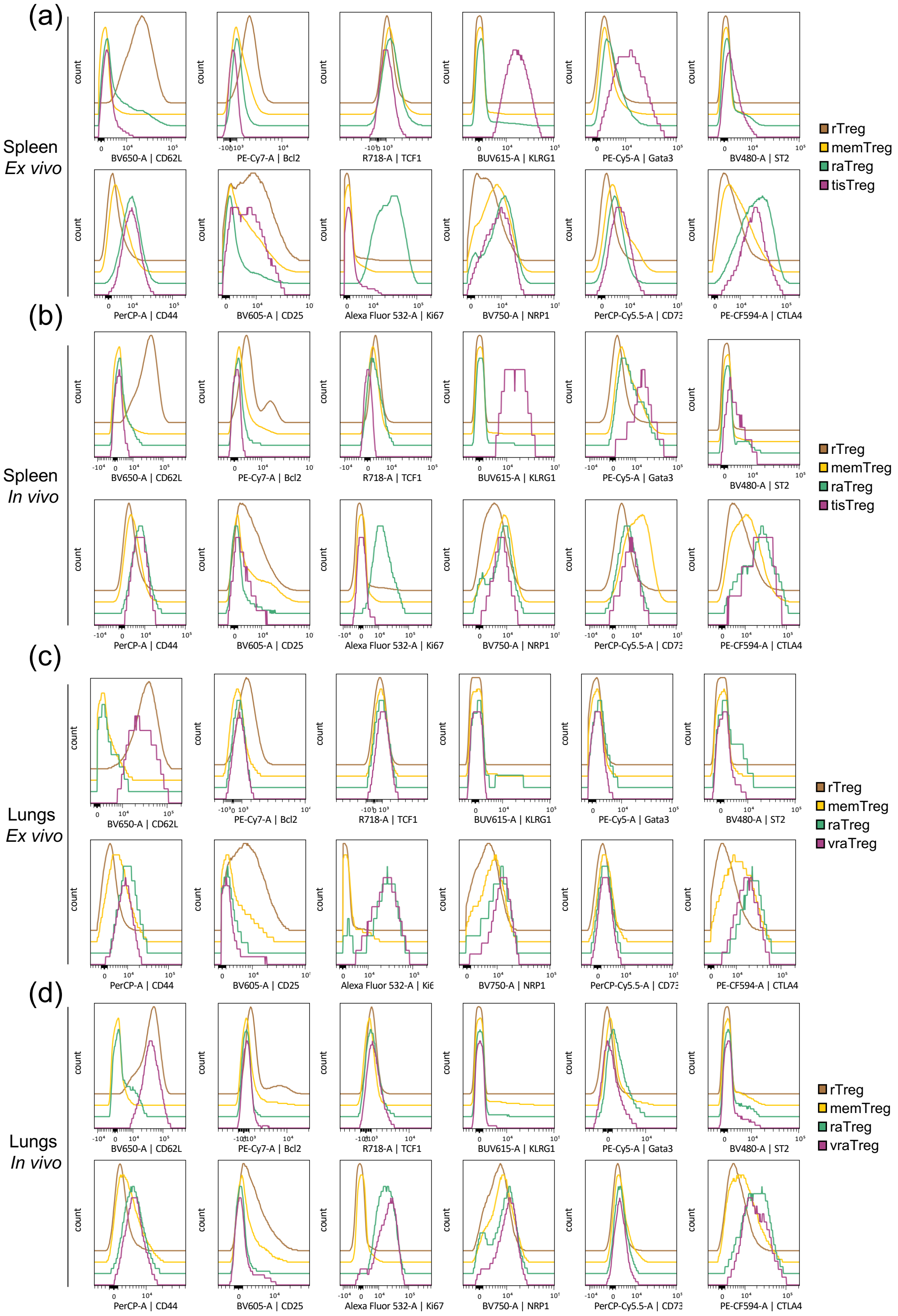

Supplementary figure 5. Phenotype of spleen and lung Treg subsets. Representative expression of characteristic and activation markers of spleen (a, b) and lung (c, d) Tregs from cells treated *ex vivo* with puromycin as in figure 3 (a, c) or mice treated *in vivo* with puromycin as in figure 1 (b, d). Expression of markers characterising resting (rTregs), memory (memTregs), recently activated (raTregs) and precursors of non-lymphoid tissue Tregs (tisTregs) are shown in the spleen. Expression of markers characterising resting (rTregs), memory (memTregs), recently activated (raTregs) and very recently activated Tregs (vraTregs) are shown in the lungs. Data are representative of at least three independent experiments (4-7 mice per group).

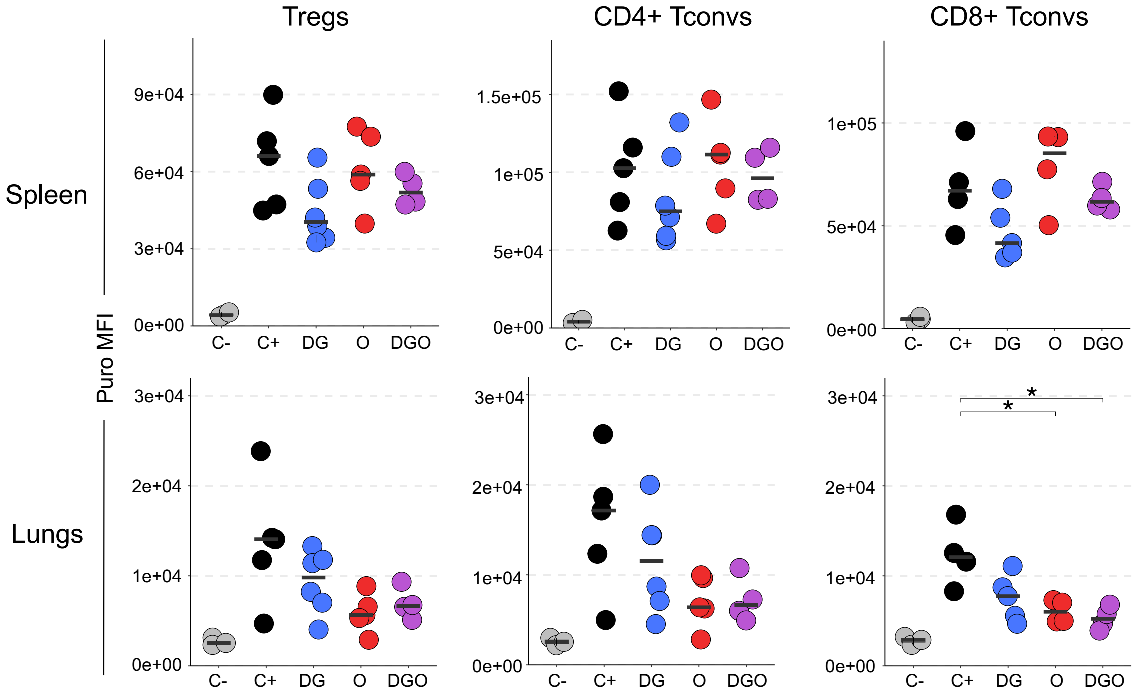

Supplementary figure 6. Puro MFI of T cells from *in vivo* SCENITH experiments. Puro MFI of spleen and lung T cells in mice treated with metabolic inhibitors as in figure 3 in an *in vivo* SCENITH assay. Each symbol represents individual mice and bars are medians. Data are from at least three independent experiments (4-6 mice per group). Statistical analysis was performed using a Wilcoxon rank sum test.

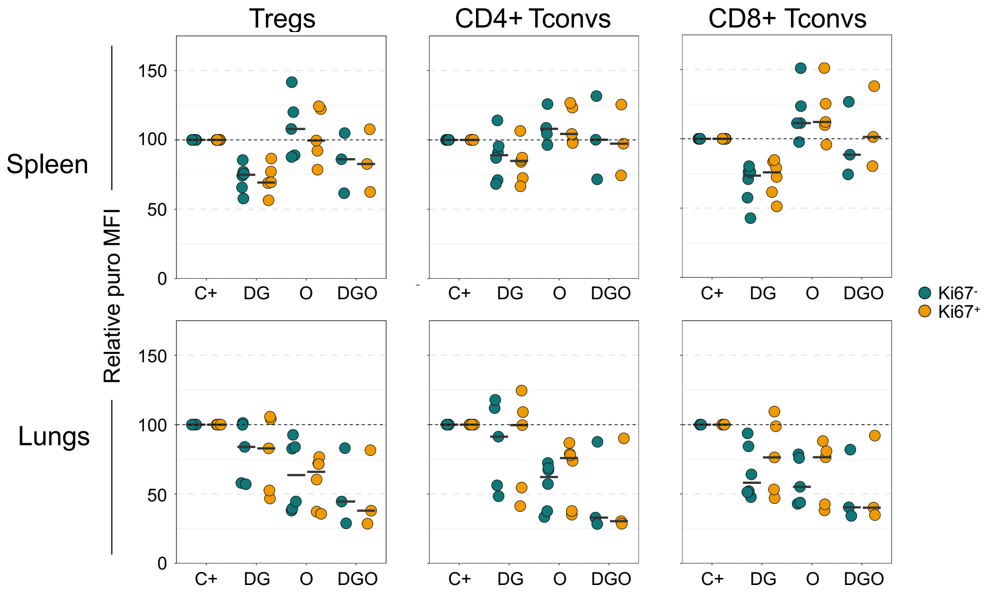

Supplementary figure 7. Puromycin staining of T cells based on cell division from *in vivo* SCENITH^{TM} experiments. Comparison of relative puro MFI between Ki67^{-} and Ki67^{+} in Tregs and Tconvs from mice treated with metabolic modulators as in figure 3 in an *in vivo* SCENITH assay. Each symbol represents individual mice and bars represent medians from at least three independent experiments (4-6 mice per group). Statistical analysis was performed using a Wilcoxon signed-rank test.

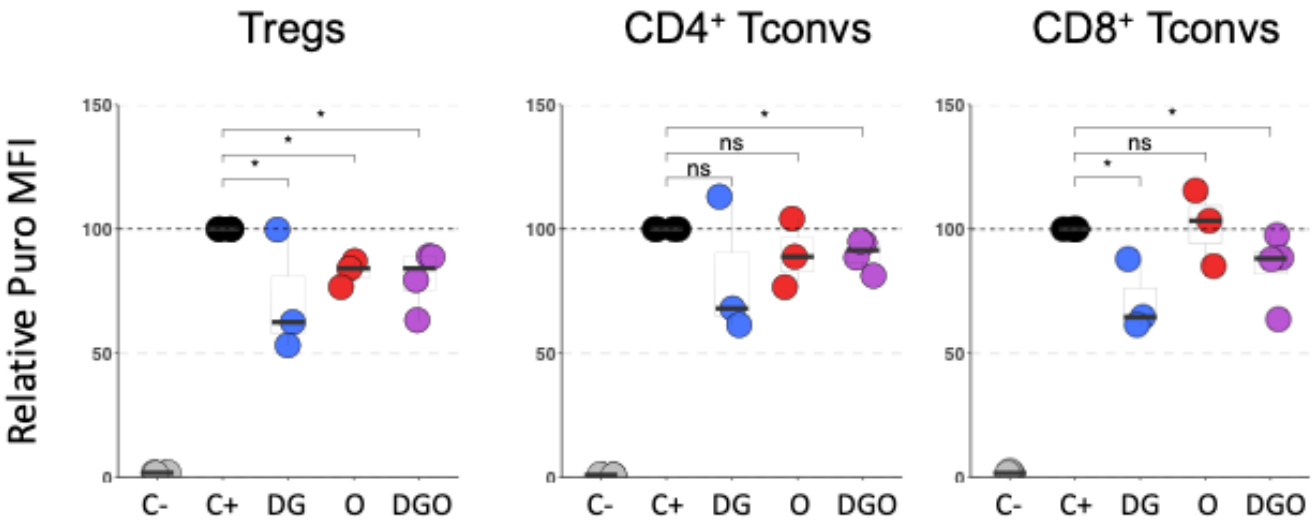

Supplementary figure 8. *In vivo* SCENITH performed in T cells from tumor draining lymph node. Mice, implanted with MCA205 12 days earlier, were treated with DG, O or both inhibitors 17- and 2-hours before euthanasia and puromycin one-hour before euthanasia. Puromycin staining was assessed in T cells derived from the draining lymph node by *in vivo* SCENITH. Each symbol represents individual mice and bars represent medians. Data are from at two independent experiments (3-4 mice per group). Statistical analysis was performed using a Wilcoxon rank sum test.

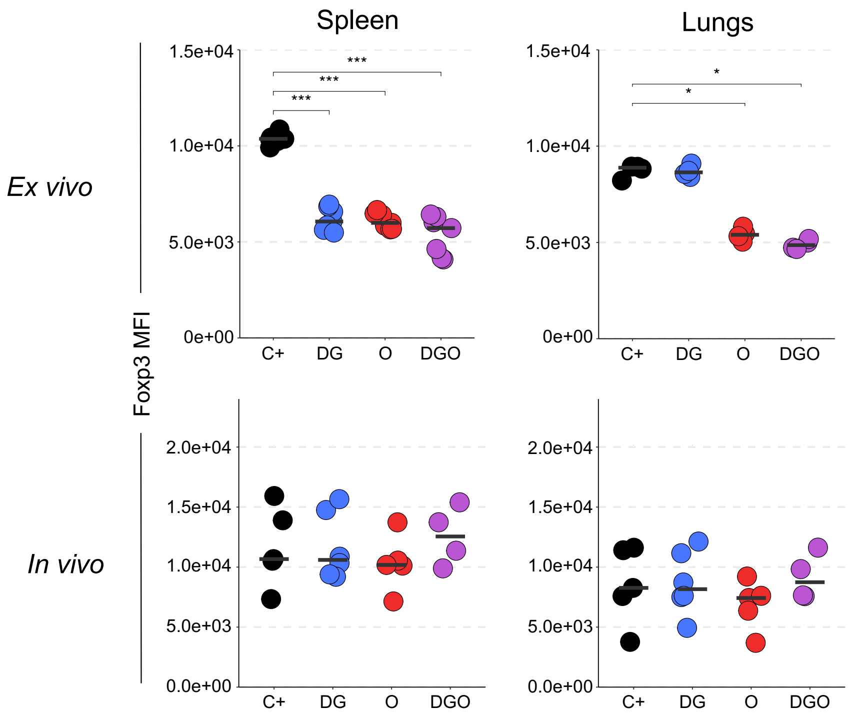

Supplementary figure 9. *In vivo* and *ex vivo* effect of 2-DG and oligomycin on Foxp3 expression of spleen and lung Tregs. Foxp3 MFI on spleen and lung Tregs from cells treated *ex vivo* with metabolic modulators as in figure 3 or mice treated *in vivo* with puromycin as in figure 1. Each symbol represents individual mice and bars are medians. Data are from at least two independent experiments (4-7 mice per group). Statistical analysis was performed using a Wilcoxon rank sum test.

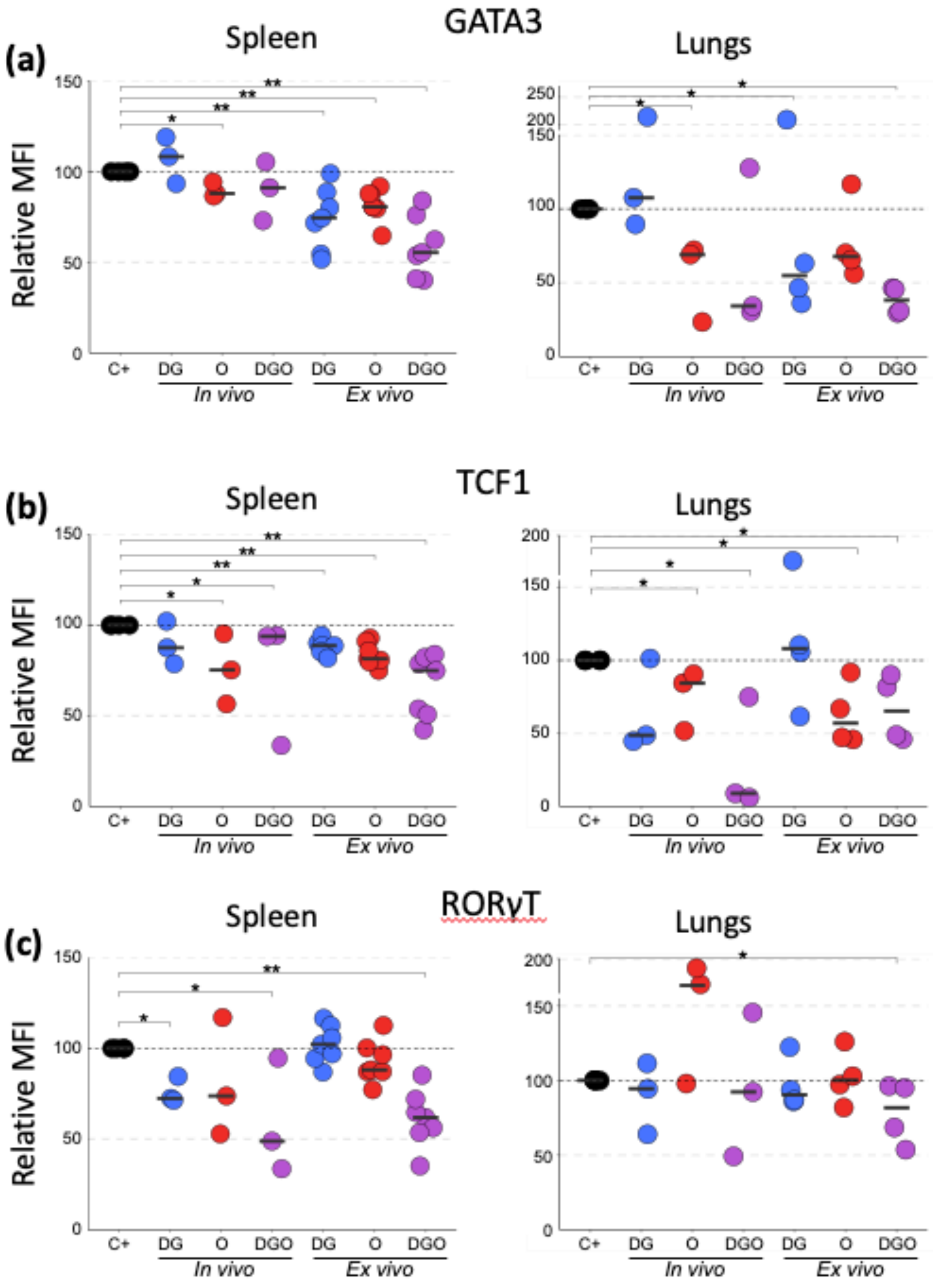

Supplementary figure 10. ***In vivo*** **and** ***ex vivo*** **effect of 2-DG and oligomycin on the expression of GATA3, TCF1 and RORγT of splenic and lung Tregs.** Relative MFI of GATA3 (a), TCF-1 (b) and RORγT (c) in splenic and lung Tregs after treating mice with the metabolic modulators and puromycin as in figure 3 (*in vivo*) or extracted cells with the metabolic modulators and puromycin as in figure 1 (*ex vivo*). Data are from at least two independent experiments (7 mice per group for *ex vivo* analysis in the spleen, 3-4 mice per group for the other conditions). Statistical analysis was performed using a Wilcoxon rank sum test.

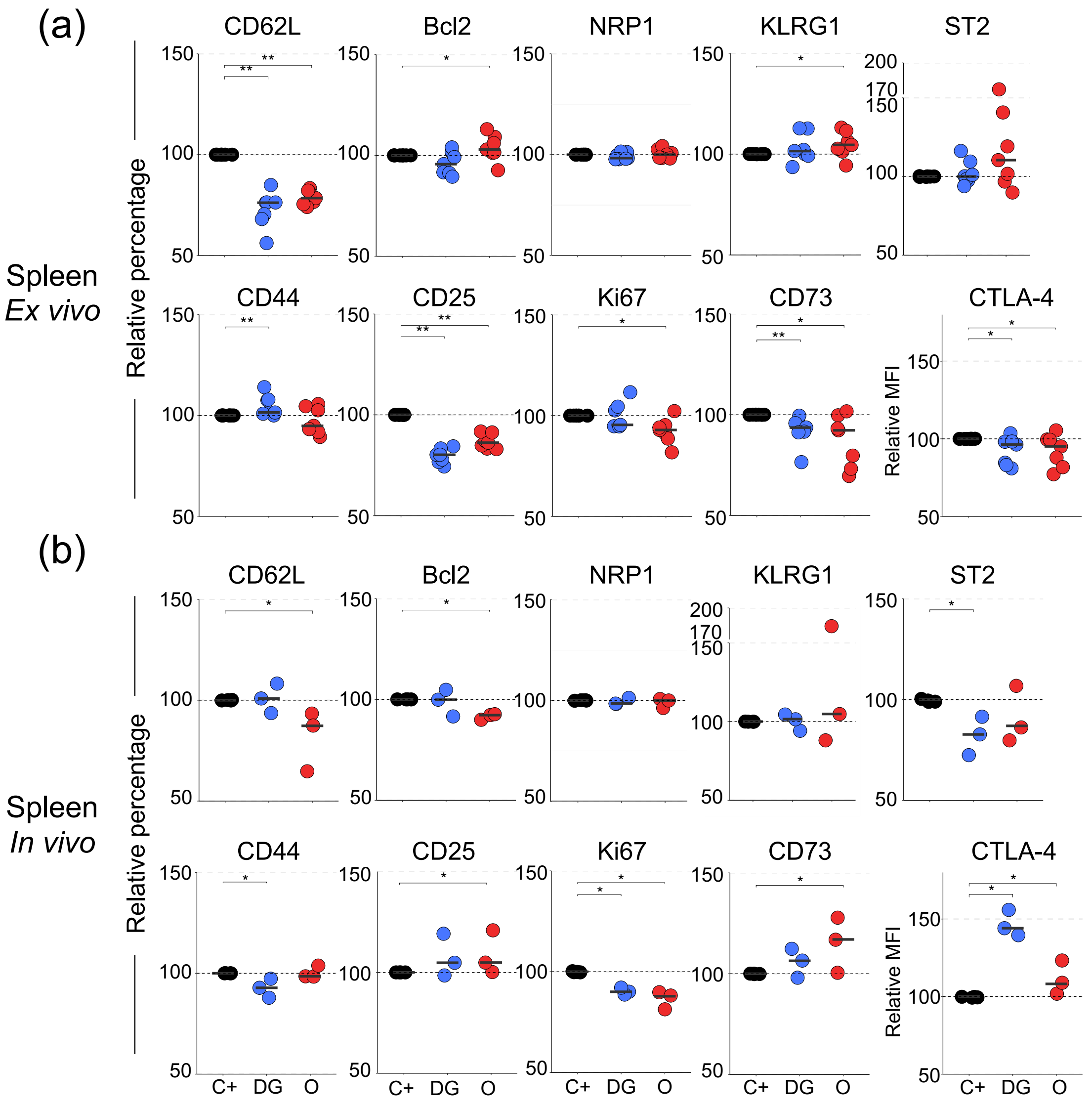

Supplementary figure 11. *In vivo* and *ex vivo* effect of 2-DG and oligomycin on the expression of characteristic and activation markers of spleen Tregs. (a, b) Relative proportion of identity and activation markers of spleen Tregs from cells treated *ex vivo* with metabolic modulators as in figure 1 (a) or mice treated *in vivo* with puromycin as in figure 3 (b). Puro MFI relative to the C+ control are shown. Data are from at least two independent experiments (7 and 3-4 mice per group for *ex vivo* and *in vivo* conditions, respectively). Statistical analysis was performed using a Wilcoxon rank sum test.

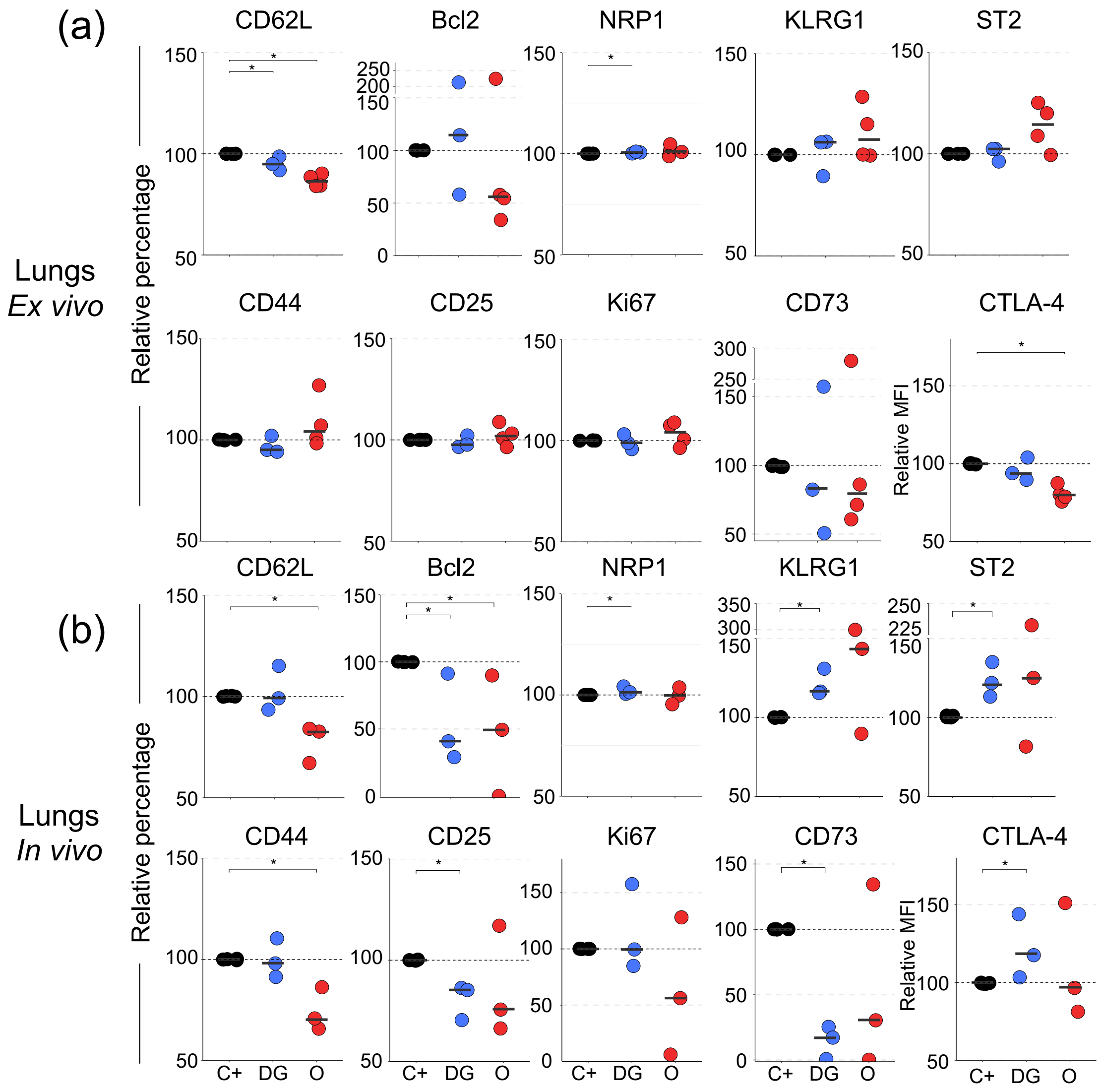

Supplementary figure 12. *In vivo* and *ex vivo* effect of 2-DG and oligomycin on the expression of characteristic and activation markers of lung Tregs. Relative proportion of identity and activation markers of lung Tregs from cells treated *ex vivo* with metabolic modulators as in figure 1 (a) or mice treated *in vivo* with puromycin as in figure 3 (b). Puro MFI relative to the C+ control are shown. Data are from at least two independent experiments (7 and 3-4 mice per group for *ex vivo* and *in vivo* conditions, respectively). Statistical analysis was performed using a Wilcoxon rank sum test.

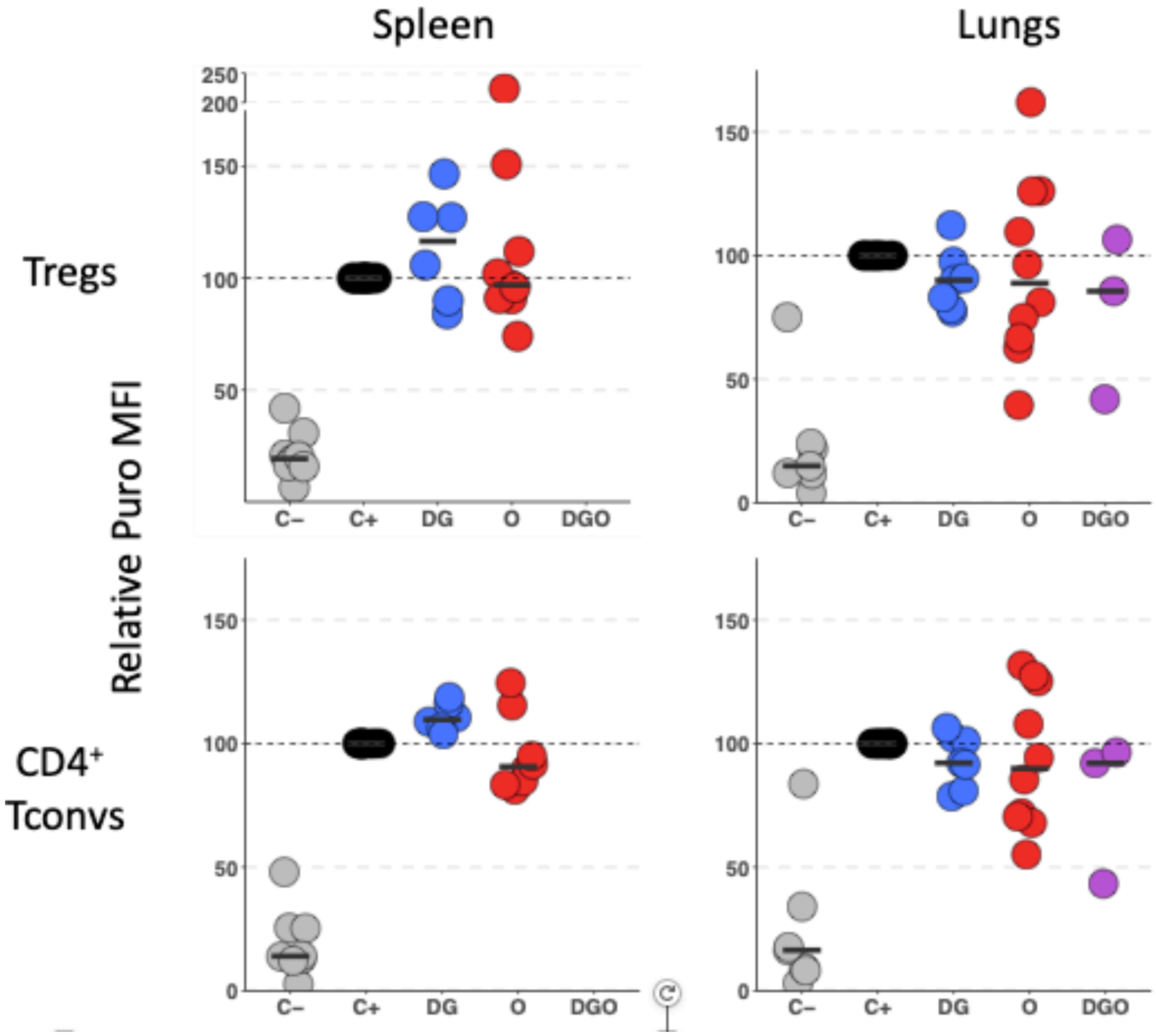

Supplementary figure 13. Metabolic profiles of CD4$^+$ T cells after only one injection of metabolic inhibitors by *in vivo* SCENITH. Mice were injected with DG, O or both 2-hours before euthanasia and puromycin one-hour before euthanasia. Puromycin staining was assessed in CD4$^+$ T cells of the spleen and lungs by *in vivo* SCENITH. Each symbol represents individual mice and bars represent medians. Data are from at least three independent experiments (6-10 mice per group except for the DGO condition that had 3 mice). Statistical analysis was performed using a *t*-test.

Supplementary table 1. Markers used to define the 4 Tregs subsets.

	rTreg	memTreg	raTreg	vraTreg	tisTreg
CD62L	High	Low	Low	High	Low
Bcl2	High	Int	Low	Low	-
TCF1	+	+	+	+	-
CD44	Low	Int	High	High	High
CTLA4	Low	Int	High	High	High
Ki67	-	-	+	+	-
KLRG1	-	-	-	-	+
GATA3	Low	Low	Low	Low	High
CD25	High	Med	Med	Med	Med
